# A pilot study of possible anti-inflammatory effects of the specific carbohydrate diet in children with juvenile idiopathic arthritis

**DOI:** 10.1186/s12969-021-00577-3

**Published:** 2021-06-10

**Authors:** Lillemor Berntson

**Affiliations:** Department of Women’s and Children’s Health, Uppsala University, Uppsala, Sweden

**Keywords:** Arthritis, Juvenile idiopathic, Pilot study, Diet therapy

## Abstract

**Background:**

To explore possible anti-inflammatory effects of the specific carbohydrate diet in children with juvenile idiopathic arthritis. This diet has shown anti-inflammatory effect in children with inflammatory bowel disease.

**Methods:**

Twenty-two patients with juvenile idiopathic arthritis (age 6.3–17.3 years), with ≤2 inflamed joints and an erythrocyte sedimentation rate < 30 mm/h, were included in this explorative study. Fifteen children completing four weeks on the diet were evaluated. A dietician introduced parents and children to the diet, and two follow-ups were performed during the intervention. Conventional laboratory tests and multiplex analyses of 92 inflammatory proteins were used. Short-chain fatty acids in faecal samples were examined.

**Results:**

The diet significantly decreased morning stiffness (*p* = 0.003) and pain (*p* = 0.048). Physical function, assessed through the child health assessment questionnaire, improved (*p* = 0.022). Arthritis improved in five of the seven children with arthritis; in those seven, multiplex analyses showed a significant decrease in nine inflammatory proteins, including TNF-alpha (*p* = 0.028), after four weeks. Faecal butyrate, analysed in all 15 participants, increased significantly (*p* = 0.020).

**Conclusion:**

The specific carbohydrate diet may have significant positive effects on arthritis in children with juvenile idiopathic arthritis, but further studies are needed.

**Clinical trials identifier:**

NCT04205500, 2019/12/17, retrospectively registered. URL:

https://register.clinicaltrials.gov

**Supplementary Information:**

The online version contains supplementary material available at 10.1186/s12969-021-00577-3.

## Introduction

Juvenile idiopathic arthritis (JIA) is an umbrella term, which describes a heterogeneous group of rheumatologic diseases that affect children; it is one of the most common chronic paediatric conditions [1]. The diagnosis encompasses seven categories, all sharing the feature of arthritis with a duration of at least six weeks and onset before the age of sixteen years [2].

The cause of the disease is considered to be multifactorial. Both the innate and the adaptive immune system have been shown to be involved in disease pathology [3, 4]. The impact of genetic factors is heterogeneous and not dominant [5]. Environmental risk factors that have been suggested to contribute to the development of JIA include use of antibiotics at early age, early weaning from breastfeeding, and delivery by caesarean section, all potentially altering the gut microbiota and intestinal immunity [6–8]. Increased gut permeability has been shown in several other inflammatory diseases and in one study on JIA, suggesting that a dysfunctional gut barrier could increase the possibility for bacteria and other substances to interplay with the immune system, leading to a breakage of T cell tolerance [9]. These factors may alter the likelihood of JIA by influencing the development of the immune system, the integrity of the intestinal mucosal barrier, and the differentiation of immune stimulatory and regulatory cells [10, 11].

As in many other autoimmune diseases, like rheumatoid arthritis (RA), the composition of the bacterial flora seems to be altered in children with JIA, though results are not consistent [12–15]. In addition to studies on environmental factors and microbiota in JIA, indicating an aberrant microbial setting, there are several studies supporting an important role for gut microbiota in relation to the immune system. The microbiome affects development of the intestinal mucosal barrier and is essential for the normal generation and maturation of gut-associated lymphoid tissue [16]. The microbiome also has an impact on production of TH17 cells [17, 18].

The occurrence and function of specific phyla, genera, or families of bacteria are being studied increasingly often and immunological processes at different levels of the intestinal canal are an important matter. Analysing the concentration of short-chain fatty acids (SCFAs) in faeces is one way to study function at the colon level. SCFAs, mainly acetate, propionate, and butyrate, are produced by bacteria in the colon through fermentation of insoluble fibres and have been shown to have profound positive immunological effects on the intestinal immune system, in particular in the case of butyrate [19].

One possible way to affect the intestinal canal is through the diet. The specific carbohydrate diet (SCD) has been shown to have beneficial effects in inflammatory bowel disease. SCD is a nutritionally balanced diet focused on removing many complex carbohydrates such as grains, dairy products except yoghurt fermented for over 24 h, vegetables rich in starch, and sugars, except monosaccharides like those found in honey. The digestion of complex carbohydrates relies on enzymes produced by the microbiota and large amounts of carbohydrates are believed to alter the microbiota. Monosaccharides, on the other hand, can be absorbed by enzymes in enterocytes and are therefore considered to have a lesser impact on the gut microbiota. Thus, the diet includes for example meat, poultry, fish, eggs, nuts, fruits, beans, peas, honey, fully fermented yogurt and hard cheese, while grains, rice, corn, potatoes, dairy products high in lactose, refined sugar and candy are excluded. Furthermore, most processed food is not allowed in SCD, as it contains emulsifiers and additives, proven to have a negative impact on the mucus layer in mouse intestines [20]. The diet has been shown to induce clinical and biochemical remission in paediatric Crohn’s disease (CD) and ulcerative colitis, but not complete healing [21–23].

The gastrointestinal tract is the largest immune system in the body, yet it is only scarcely studied in JIA. Great advances have been made in treatment of rheumatologic diseases, but not even biological agents lead to full response rates in JIA [24, 25]. The aim of this study was to explore if SCD would have an anti-inflammatory effect in children with JIA and thus provide a potential complementary treatment option.

## Methods and materials

### Cohort description and clinical variables

The author recruited children and teenagers with JIA, classified in accordance with the International League of Associations for Rheumatology’s criteria, at the paediatric rheumatology unit of Uppsala University Children’s Hospital in Sweden, from September 2017 to September 2019 [2]. Further inclusion criteria were that patients had to be on stable treatment – i.e., there had been no change in medical treatment with disease-modifying anti-rheumatic drugs (DMARDs) or biological DMARDs (bDMARDs) during the preceding twelve weeks – with a mild to moderate disease activity, no more than two active joints at inclusion, and an erythrocyte sedimentation rate (ESR) of no more than 30 mm/h. Fulfilment of the criteria for not being in remission, as described by the American College of Rheumatology, was also required [26]. The medical treatment of any kind, during the observation period of one month had to be stable, in case this was not possible, the child was excluded. Children could only be included if parents and children were strongly motivated to try a dietary intervention as a complementary treatment. Inclusion in the study was based on voluntariness: the majority of families had read about the study and spontaneously asked for the child to be included; in some cases, inclusion was offered. Children with any gastrointestinal complaints were investigated and faecal calprotectin < 100 g/L was required for inclusion. The primary goal was for the child to pursue the diet for four weeks or longer. Before inclusion, the families each received a recipe booklet, a list of allowed products, and a list of recommendations. An initial telephone appointment with a dietician was obligatory. A visit to the office of the paediatric rheumatology clinic was performed for inclusion.

After the inclusion visit, families were instructed to get familiar with what food to eat and what to avoid during a “learning period” of two weeks at most. After this two-week period, the participants were instructed to follow the SCD diet strictly for at least four weeks, with a follow-up visit after two and four weeks on SCD. The families were also instructed to fill out a food diary during three days at the very end of the four-week period. At follow-ups, clinical examinations were performed, with weight measured and faecal, urine, and blood samples collected. The child health assessment questionnaire (CHAQ) was filled out [27]. Throughout the trial, the families had regular contact with and access to the dietician, by email and telephone, and also the physician, by email.

Assessments of CHAQ, juvenile arthritis disease activity score (JADAS27), morning stiffness in minutes, and pain visual analogue scale (VAS) (0–10 cm) were made at inclusion and after two and four weeks of treatment. Levels of SCFAs in faecal samples from the same occasions were analysed using a high-performance liquid chromatography machine, Agilent technology 1100 series (Agilent Technologies, Inc., Santa Clara, USA). JADAS27 (0–57) is a validated composite disease activity score often used for monitoring patients with JIA. It comprises 1) the number of active joints (0–27), 2) patient global assessment VAS (0–100 mm), 3) physician global assessment VAS (0–100 mm), and 4) ESR normalised to a scale 0–10 [28]. Since a low disease activity may be difficult to measure with conventional laboratory tests in JIA, a multiplex analysis of 92 inflammation-associated proteins in plasma before and after treatment was included (Proseek Multiplex Inflammation; Olink Bioscience, Uppsala, Sweden) [29]. Arthritis was assessed at clinical examination, not using ultrasound at inclusion or follow-ups.

The endpoints of this study were to examine whether SCD for four weeks would affect arthritis, clinical pain VAS (0–100 mm), morning stiffness (minutes), physical function assessed through CHAQ, global assessment VAS by patient/parent (0–100 mm), ESR, CRP, or SCFAs in faecal samples. A multiplex system for analysis of 92 chemokines involved in inflammation was added when the fifteen patients had been included.

### Statistical analysis

The Wilcoxon signed-rank test was used as the non-parametric test to estimate significance of differences in clinical and laboratory variables before and during treatment with SCD. The Hodges-Lehmann related sample analysis was used to estimate the confidence interval of the estimated median values. Adjustment for multiple comparisons was performed using the Benjamini-Hochberg method, with a false discovery rate of 10%. All tests were considered to be significant at *p* < 0.05 and all statistical analyses were performed using IBM SPSS Statistics for Windows, version 25 (IBM Corp., Armonk, NY, USA).

## Results

### Participants and clinical variables

Twenty-two children with different categories of JIA were recruited to this trial and fifteen of them completed the four-week intervention; in three children, the follow-up was performed after five weeks. The JADAS27 value in the fifteen children at inclusion was median (Md) 5.2 (3.4–9.7). Three of the children with the lowest JADAS27 belonged to the oligoarticular persistent category and JADAS27 was 2.2,;2.2 and 3.4, respectively – thus, they were in a low to moderate disease activity state according to Consolaro et al. [28] One child with polyarticular RF negative JIA was in an inactive disease state based on the JADAS27, with a value of 0.3 [28], but she had periods of morning stiffness ≥15 min and wanted to stop treatment with methotrexate. According to the American College of Rheumatology remission criteria, she was not in remission [26]. None of the children had received intra-articular steroid injections within the last eight weeks before inclusion. Demographic data are presented in Table 1.

**Table 1:** Clinical characteristics of fifteen children with juvenile idiopathic arthritis, treated with specific carbohydrate diet for four weeks

Patient ID	Gender	ILAR category^a^	Age at onset, years	Age at inclusion, years	Clinical features at inclusion	Treatment during study	ANA^b^	HLA-B27^c^
1	F	Oligo pers	16.0	17.3	Arthritis, one knee	None	Neg	Pos
2	F	ERA	14.0	14.3	Arthralgia + morning stiffness	NSAID	Neg	Pos
3	M	Oligo pers	2.9	9.0	Arthritis, one knee	None	Pos	na^d^
4	F	Juv psoriatic	13.6	16.7	Morning stiffness	NSAID	Neg	na^d^
5	F	Poly RF^−^	13.6	16.5	Morning stiffness	Methotrexate	Neg	Neg
6	F	Oligo ext	3.0	13.8	Arthritis, one knee + uveitis	Infliximab	Neg	na^d^
7	F	Oligo pers	3.2	11.7	Pain + stiffness after physical activity, post uveitis	Abatacept + Methotrexate	Neg	na^d^
8	F	Poly RF^−^	8.0	10.8	Arthritis, one ankle	Abatacept	Pos	na^d^
9	M	Poly RF^−^	13.7	17.1	Arthritis, one ankle, one elbow	Methotrexate	Pos	Neg
10	F	Oligo pers	10.8	14.4	Inflammatory back pain	Etanercept + Methotrexate	Neg	Neg
11	F	Oligo pers	5.6	9.5	Morning stiffness, recurrent joint injections	Methotrexate	Pos	na^d^
12	F	Oligo pers	3.7	11.2	Joint pain after physical activity, post uveitis	Infliximab Methotrexate	Pos	na^d^
13	M	Oligo pers	1.5	14.6	Arthritis, one knee	Methotrexate	Neg	Neg
14	F	Oligo pers	5.6	6.3	Morning stiffness, pain	Methotrexate	Neg	na^d^
15	M	ERA	9.4	10.9	Arthritis, one SI^e^-joint	None	na^d^	Pos

^a^ILAR, International League of Associations for Rheumatology; oligo pers., oligoarticular persistent; ERA, enthesitis-related arthritis; juv psoriatic, juvenile psoriatic; poly RF^−^, polyarticular rheumatoid factor negative; oligo ext., oligoarticular extended

^b^ANA, antinuclear antibodies

^c^the human leukocyte antigen B27

^d^na, not analysed

^e^SI, sacroiliac

Six families dropped out before two weeks of the study due to lack of motivation; in four, the child turned out not to be motivated enough, and in two, the parents were not motivated enough. In one family, an acute psychosocial situation stopped participation. Two of the participants did not take part in the two-week visit, because they lived far away from the clinic. Eleven (73%) of the participants were girls and four were boys. The median age at inclusion was 13.8 years (IQR: 10.8–16.5) and median disease duration was 3.1 years (IQR: 1.7–7.6). Seven of the fifteen participants had active arthritis at time of inclusion. Faecal calprotectin levels were 0–69 g/L (min–max) at inclusion (reference value 50 g/L). Weight decreased with a median of 2% during the one-month intervention. Five patients were on a stable treatment with methotrexate before and during the month of intervention, three were on TNF inhibitors (two in combination with methotrexate), two were on abatacept (one in combination with methotrexate), two were treated with NSAIDs, and three received no treatment.

Five of the seven children with arthritis at inclusion did not have any clinical signs of arthritis after four to five weeks of SCD. In two children, one with enthesitis-related arthritis and one with juvenile psoriatic arthritis, the inflammatory activity increased very shortly after inclusion in the study. One of them also developed a virus infection after three weeks on SCD and the JIA had worsened after five weeks on SCD.

**Fig. 1:**
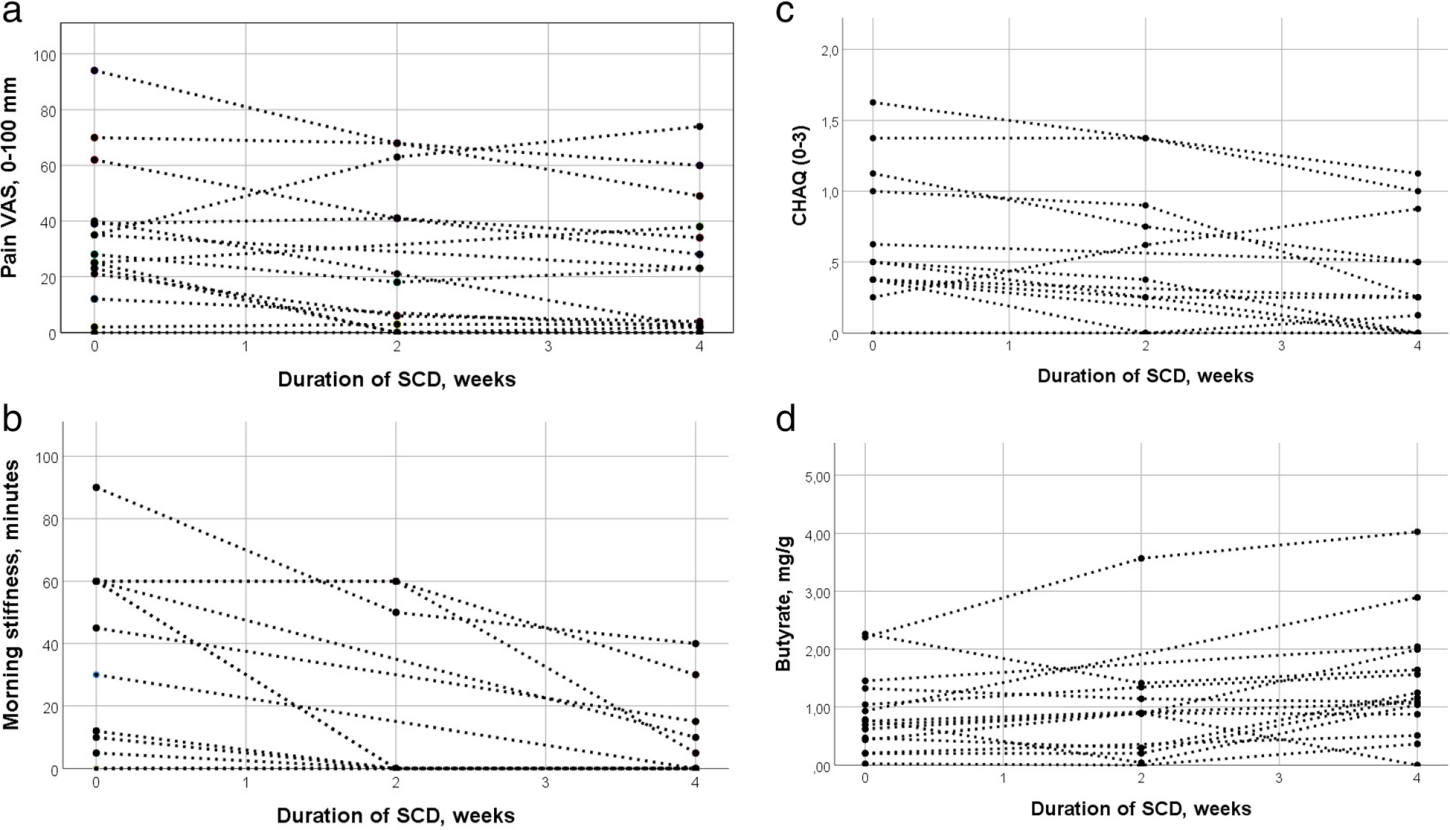
**a**–**d** Levels of pain on visual analogue scale (0–100 mm) (**a**); Morning stiffness (minutes) (**b**); Child health assessment questionnaire, CHAQ (0–3) (**c**); Concentration of butyrate (mg/g) in faecal samples (**d**). All at inclusion and after two and four weeks of specific carbohydrate diet. *Wilcoxon matched-pair signed rank analysis comparing levels at inclusion with levels after four weeks of specific carbohydrate diet

Pain VAS decreased significantly from Md 28 mm (IQR: 2.1–4.0) to Md 23 mm (IQR: 2.0–3.8) (Fig. 1), as did morning stiffness, from Md 30 min (IQR: 0–60) to Md 0 min (IQR: 0–10) (Fig. 1). CHAQ improved significantly during the study period from Md 0.4 (IQR: 0.2–1.0) to Md 0.2 (IQR: 0–0.5) (Fig. 1). JADAS27 improved, but not significantly, *p* = 0.065. Patient global assessment VAS improved, but not significantly, *p* = 0.069. Butyrate in faecal samples in the whole cohort increased significantly during the diet period (Fig. 1), while propionate and total levels of SCFAs increased non-significantly, presented in Table 2.

**Table 2:** Levels of short chain fatty acids (SCFAs) in faecal samples before, compared with at four weeks of treatment with specific carbohydrate diet (SCD) in fifteen patients with juvenile idiopathic arthritis

SCFA mg/g^a^	At inclusion Md (IQR)^b^	At 4 weeks of SCD	Median difference	Confidence interval^*^	*p*-value ^**^
Butyrate	0.7 (0.4–1.3)	1.2 (0.9–2.0)	0.5	0.08–1.0	0.02
Propionate	0.9 (0.5–1.0)	0.9 (0.6–1.3)	0.2	(− 0.01)–(0.4)	0.06
Acetate	3.0 (2.2–3.7)	3.7 (2.1–5.3)	0.4	(−0.5)–(1.1)	0.3
Valerate	0.1 (0.04–0.2)	0.1 (0.06–0.2)	0.02	(−0.05)–(0.1)	0.5
Total	5.4 (3.6–6.8)	6.6 (4.2–8.5)	1.1	(−0.2)–(2.4)	0.07

^a^mg = milligram; g = gram

^b^Md = median; IQR = interquartile range

^*^Hodges-Lehmann related sample analysis

^**^Wilcoxon matched-pair signed rank analysis

### Biomarkers

There was no significant difference in inflammatory blood tests between baseline and week four/five (data not shown), in the total cohort, but only four participants had an ESR > 10 mm/h at inclusion. In the paired analyses of 92 inflammatory proteins in the whole group of 15 patients, a significant decrease in 13 chemokines was found (Additional Table 1), but after adjustment for multiple comparisons, only the decreases in SCF, IL-10B and CX3CL1 remained significant. In the seven patients with active arthritis at inclusion, the analyses of 92 inflammatory proteins with the described multiplex system showed a significant decrease in nine chemokines, TNF-alpha, TRAIL, MCP-1, CX3CL1, ADA, IL10RA, IL10RB, SCF, and uPA, presented in Table 3, but none of them remained significant after adjustment for multiple comparisons. Five of the 13 chemokines that decreased in the whole group decreased in the 7 children with arthritis as well, and three of those chemokines were the ones for which decreases remained significant after adjustment for multiple comparisons: CX3CL1, SCF and IL-10RB. The level of TNF-alpha decreased significantly only in the group with arthritis at inclusion; MCP-1 decreased significantly in both the group with arthritis and the whole group, but the decrease did not remain significant after adjustment for multiple comparisons. No significant *increase* in any chemokine was found in any of the analyses.

**Table 3:** Levels of inflammatory proteins in paired samples before, compared with at four weeks of specific carbohydrate diet treatment in the seven patients with arthritis at inclusion

Chemokine pg/ml^a^	At inclusion Md (IQR)^b^	At 4 weeks Md (IQR)^b^	Median difference	Confidence interval^*^	p-value^**^
TNF-alpha	1.8 (1.2–4.1)	1.4 (1.0–3.9)	−0.3	(− 0.5)–(− 0.1)	0.03
TRAIL	6.8 (6.7–6.9)	6.7 (6.4–6.9)	−0.2	(−0.4)–(− 0.04)	0.03
MCP-1	11.2 (10.6–11.5)	10.5 (10.4–10.8)	−0.5	(−1.1)–(− 0.01)	0.03
CX3CL1	4.8 (4.5–4.9)	4.4 (4.0–4.6)	−0.3	(−0.4)–(− 0.08)	0.02
ADA	2.1 (1.7–2.9)	1.7 (1.4–2.6)	−0.4	(−0.9)–(− 0.08)	0.02
IL10RA	0.3 (0.1–0.3)	0.05 (−0.07–0.18)	−0.1	(− 0.3)–(− 0.04)	0.02
IL10RB	5.1 (4.9–5.2)	4.9 (4.8–5.0)	−0.2	(−0.5)–(− 0.07)	0.03
SCF	8.6 (8.4–8.9)	8.2 (7.6–8.5)	−0.5	(−1.0)–(− 0.2)	0.02
uPA	9.8 (9.3–9.8	9.4 (9.3–9.7)	−0.1	(−0.4)–(− 0.02)	0.02

^a^pg/ml = picogram/millilitre

^b^Md = median; IQR = interquartile range

^*^Hodges-Lehmann related sample analysis

^**^Wilcoxon matched-pair signed rank analysis

## Discussion

Diet affects the composition and function of the microbiota, which may have implications on health [30]. In this study, SCD as a complementary treatment in patients with JIA resulted in a significant improvement in morning stiffness, pain, and physical function. A significant decrease in inflammatory proteins was also found in the seven children with arthritis at inclusion. An increase in faecal butyrate concentration from four weeks of SCD in fifteen children coincided at a group level with improvement in clinical variables.

Participants in this study had a low to medium disease activity at inclusion; approximately half of them had morning stiffness and pain as remaining complaints from the disease, in spite of medical treatment. The clinical assessments of pain, morning stiffness, global assessment VAS, and physical function improved already after two to three weeks in the majority of participants and pointed to a positive effect from SCD. Disease activity according to JADAS27 decreased, but not significantly – which was not surprising given the low JADAS27 at inclusion. The inflammatory activity in two patients increased very shortly after inclusion in the study, and the arthritis in those two children did not respond to treatment. In the remaining five with arthritis at inclusion, no clinical sign of arthritis could be found after four/five weeks on SCD.

Laboratory analysis results of blood samples did not change significantly during the study period, but only four of the children had an ESR > 10 mm/h at inclusion. In the multiplex analyses in the seven patients with arthritis at inclusion, a decrease in nine chemokines was found, but the majority of those have not been studied in JIA earlier. It is difficult to draw any conclusion from the results, except that there was a decrease in TNF-alpha and MCP-1 at the group level. Five of the nine chemokines that decreased significantly in the seven patients decreased significantly also in the whole group of fifteen patients, but the decreases remained significant after correction for multiple analyses only for three chemokines: CX3CL1, SCF and IL-10RB. Both MCP-1 and CX3CL1 have shown significant chemoattractant roles in recruiting inflammatory cells to synovial joints in RA and have both been associated with disease activity scores in RA [31, 32]. At a group level, the laboratory results from a multiplex system in the seven children with arthritis at inclusion, raised the hypothesis that four weeks of SCD possibly had a positive impact on the inflammatory process, but not in every individual. One could also speculate that the impact in two of the children was too weak and the intervention may have been too short to affect the inflammatory process.

The results of a significant increase in butyrate and an increasing, yet non-significant, level of SCFAs in faeces are not surprising, since fibres and starches found in fruits and vegetables are vital substrates for the production of butyrate and other SCFAs. The SCFAs are proven to have many beneficial functions, contributing to an anti-inflammatory state of the intestine. Several studies have shown that these microbial metabolites, especially butyrate – in addition to being an energy substrate for the epithelial cells of the colon – have profound effects on T cells, directly and indirectly regulating their differentiation [19, 33]. New findings also suggest that butyrate can suppress arthritis by influencing the development and function of regulatory B cells in mice [34].

SCD contains large amounts of dietary fibres. Low dietary fibres may cause catabolism of the mucous layer, leading to increased permeability and allowing increased contact between luminal bacteria and the epithelium [35]. The composition of the bacterial flora, the diet of the host, and the transit time in the gut are some of the factors influencing the production of SCFAs. While the butyrate level increased in faecal samples from the participants, it is not known if butyrate is involved in regulation of inflammation in children with JIA; most likely, it plays an anti-inflammatory role.

This study on SCD comprised only fifteen patients and the arthritis was not verified by ultrasound, which are its major weaknesses. Also, children with different categories of the disease, on different medical treatments, were included, which may have confounded interpretation of results. It was a challenge to coordinate inclusion of a patient with a period of three months of a stable, low to median inflammatory state. Also, it would have been preferable to have a control group, which was difficult to organize in practice.

The author can only speculate that elimination of processed food, additives, and emulsifiers, and restriction of carbohydrates and dairy products play an immunological role in JIA. Processed food often contains high amounts of exogenous advanced glycation end products (AGEs), which are common in food products that have been heated. Exogenously added AGEs have been shown in animal studies to affect immune and epithelial cells by activating the receptors for AGEs in various types of cells, such as immune cells, endothelial cells, myocytes, and neurons, but studies in humans have not come that far [36]. High-fructose corn syrup (HFCS) is a popular sweetener in the food industry, for example in soda. High consumers of sodas have been shown to have an increased risk of arthritis in adults compared with low consumers [37]. HFCS is decreased in SCD compared with in a conventional diet; there is currently a lack of knowledge about the occurrence of AGEs.

The results from this study indicate that a diet like SCD may be of benefit in children with JIA and that studies on the immunological influence from diet in such children may potentially open up a new field of research.

## Conclusion

Making the home-cooked meals required in SCD was a challenge for many of the families, but the fairly rapid improvement in the majority of the children motivated both parents and children. A strength of the study was that the SCD is well-described and studied in children with inflammatory conditions in the digestive tract, as one of two diets studied in paediatric IBD. The results from this study suggest that specific carbohydrate diet may provide a promising complementary treatment modality for children with JIA. Further studies are needed to understand which children with JIA may benefit from SCD, how the diet affects the immune system, and how long-lasting any effects are.

## Supplementary Information

**Additional file 1: Table S1.** Presents levels of inflammatory proteins in paired samples before, compared with at four weeks of SCD treatment in the whole group of fifteen patients with JIA.

**Additional file 2: Table S2.** Presents the clinical characteristics and medical treatment in fifteen children with juvenile idiopathic arthritis, before inclusion in the study.

## Data Availability

The datasets generated and/or analyzed during the current study are not publicly available for ethical reasons, as well as privacy reasons, but are available from the author on reasonable request.

## Abbreviations

JIA: juvenile idiopathic arthritis
RA: rheumatoid arthritis
SCFAs: short chain fatty acids
SCD: specific carbohydrate diet
CD: Crohn’s disease
DMARD: Disease-modifying anti-rheumatic drug
bDMARD: Biological DMARD
ESR: erythrocyte sedimentation rate
CHAQ: child health assessment questionnaire
JADAS: juvenile arthritis disease activity score
VAS: visual analogue scale
ADA: adenosine deaminase
IL: interleukin
AGE: advanced glycation end products
HFCS: high-fructose corn syrup
