# Supplementary material for: A pilot study of possible anti-inflammatory effects of the specific carbohydrate diet in children with juvenile idiopathic arthritis
Source: Pediatr Rheumatol Online J. 2021 Jun 10;19:88. doi: 10.1186/s12969-021-00577-3 (PMC8194161; doi:10.1186/s12969-021-00577-3)
Supplement: Supplementary file 1 — Additional file 1: Table S1. Presents levels of inflammatory proteins in paired samples before, compared with at four weeks of SCD treatment in the whole group of fifteen patients with JIA. [file 12969_2021_577_MOESM1_ESM.docx]

Additional Table 1. Levels of inflammatory proteins from a multiplex panel in paired samples before, compared with at four weeks of SCD^a^ treatment, in the whole group of fifteen patients with JIA.

| Chemokine  pg/ml^b^ | At inclusion  Md (IQR)^c^ | At 4 weeks  Md (IQR)^c^ | Median difference | Confidence interval^*^ | p-value^**^ |
| --- | --- | --- | --- | --- | --- |
|  |  |  |  |  |  |
| SCF | 8.6 (8.4–8.9) | 8.2 (7.9–8.5) | -0.4 | (-0.6)–(-0.2) | < 0.001 |
| IL-10RB | 5.1 (4.9–5.2) | 4.9 (4.8–5.0) | -0.2 | (-0.2)–(-0.08) | < 0.001 |
| CX3CL1 | 4.8 (4.5–4.9) | 4.6 (4.4–4.7) | -0.2 | (-0.3)–(-0-08) | < 0.01 |
| IL-18R1 | 7.2 (7.0–7.6) | 7.1 (6.8–7.4) | -0.2 | (-0.3)–(-0.05) | < 0.01 |
| HGF | 7.1 (6.9–7.5) | 6.9 (6.8–7.4) | -0.2 | (-0.3)–(-0.07) | 0.01 |
| IL-12B | 4.9 (4.7–5.2) | 4.8 (4.4–5.1) | -0.2 | (-0.3)–(-0.05) | 0.01 |
| MCP-1 | 11.1 (10.8–11.4) | 10.8 (10.5–11.1) | -0.3 | (-0.6)–(-0.06) | 0.01 |
| ADA | 2.1 (2.0–2.6) | 2.1 (1.7–2.4) | -0.2 | (-0.4)–(-0.08) | 0.02 |
| FGF-5 | -0.3 ((-0.3)–(-0.2)) | -0.3 ((-0.4)–(-0.2)) | -0.1 | (-0.1)–(-0.01) | 0.02 |
| CST5 | 4.1 (3.9–4.5) | 3.9 (3.7–4.3) | -0.2 | (-0.3)–(-0.02) | 0.02 |
| CD40 | 10.2 (9.9–10.5) | 9.8 (9.6–10.4) | -0.3 | (-0.5)–(-0.03) | 0.03 |
| FGF-23 | 1.5 (1.2–1.9) | 1.5 (1.1–1.7) | -0.1 | (-0.3)–(-0.02) | 0.03 |
| Flt3L | 8.1 (7.9–8.3) | 7.9 (7.7–8.2) | -0.2 | (-0.3)–(-0.02) | 0.04 |
|  |  |  |  |  |  |
| ^a^SCD = specific carbohydrate diet  ^b^pg/ml = picogram/millilitre.  ^c^Md = median; IQR = interquartile range.  ^*^Hodges-Lehmann related sample analysis.  ^**^Wilcoxon matched-pair signed-rank analysis. | | | | |  |
