# Supplementary material for: A pilot study of possible anti-inflammatory effects of the specific carbohydrate diet in children with juvenile idiopathic arthritis
Source: Pediatr Rheumatol Online J. 2021 Jun 10;19:88. doi: 10.1186/s12969-021-00577-3 (PMC8194161; doi:10.1186/s12969-021-00577-3)
Supplement: Supplementary file 2 — Additional file 2: Table S2. Presents the clinical characteristics and medical treatment in fifteen children with juvenile idiopathic arthritis, before inclusion in the study. [file 12969_2021_577_MOESM2_ESM.docx]

Additional Table 2. Clinical characteristics and medical treatment in fifteen children with

juvenile idiopathic arthritis, before inclusion in the study.

| Patient ID | ILAR category^a^ |  | Treatment during study | Duration of DMARD^b^ or bDMARD^c^ treatment before inclusion, months | | | Number of joint injections during the current treatment, before inclusion |
| --- | --- | --- | --- | --- | --- | --- | --- |
| 1 | Oligo pers |  | None |  | - | |  |
| 2 | ERA |  | NSAID^d^ |  | - | |  |
| 3 | Oligo pers |  | None |  | - | |  |
| 4 | Juv psoriatic |  | NSAID^d^ |  | - | |  |
| 5 | Poly RF^-^ |  | Methotrexate |  | 22 | | 1 |
| 6 | Oligo ext |  | Infliximab |  | 7 | | 3 |
| 7 | Oligo pers |  | Abatacept + Methotrexate |  | Abatacept 24, Methotrexate 10^e^ | | 2 |
| 8 | Poly RF^-^ |  | Abatacept |  | 5 months | | 1 |
| 9 | Poly RF^-^ |  | Methotrexate |  | 3 months | | 1 |
| 10 | Oligo pers |  | Etanercept + Methotrexate |  | Etanercept 13, Methotrexate 18 | | 2 |
| 11 | Oligo pers |  | Methotrexate |  | 6 | | 3 |
| 12 | Oligo pers |  | Infliximab Methotrexate |  | 48 | | 0 |
| 13 | Oligo pers |  | Methotrexate |  | | 52 | 5 |
| 14 | Oligo pers |  | Methotrexate |  | | 8 | 3 |
| 15 | ERA |  | None |  | | - |  |

^a^ILAR, International League of Associations for Rheumatology; oligo pers, oligoarticular persistent; ERA, enthesitis-related arthritis; juv psoriatic, juvenile psoriatic; poly RF^-^, polyarticular rheumatoid factor negative; oligo ext, oligoarticular extended. ^b^DMARD, disease-modifying anti-rheumatic drug; MTX, methotrexate. ^c^bDMARD, biological DMARD; ETN, etanercept. ^d^NSAID = nonsteroidal anti-inflammatory drug ^e^at the current dose
